# SORBS2 as a molecular target for atherosclerosis in patients with familial hypercholesterolemia

**DOI:** 10.1186/s12967-022-03381-z

**Published:** 2022-05-19

**Authors:** Ming-Ming Liu, Jia Peng, Yuan-Lin Guo, Cheng-Gang Zhu, Na-Qiong Wu, Rui-Xia Xu, Qian Dong, Chuan-Jue Cui, Jian-Jun Li

**Affiliations:** Cardiovascular Metabolic Center, State Key Laboratory of Cardiovascular Diseases, Fu Wai Hospital, National Clinical Research Center for Cardiovascular Diseases, Chinese Academy of Medical Sciences and Peking Union Medical College, No. 167 BeiLiShi Road, XiCheng District, Beijing, China

**Keywords:** SORBS2, Oxidized low density lipoprotein, NLRP3 inflammasome, ROS, Cholesterol efflux

## Abstract

**Background:**

Familial hypercholesterolemia (FH) is a metabolic disease in which patients are prone to develop premature atherosclerosis (AS). Sorbin and SH3 Domain Containing 2 (SORBS2) is known to play a role in coronary heart disease (CHD). However, the mechanism underlying SORBS2 involvement in the development of hypercholesterolemia remains unknown. Here, we investigated the effects of SORBS2 on inflammation and foam cell formation and its underlying mechanisms.

**Methods:**

Using Bioinformatics analysis, we established that SORBS2 is upregulated in patients with FH. Circulating concentrations of SORBS2 were measured using ELISA kit (n = 30). The association between circulating SORBS2 levels and inflammatory factors or lipid indexes were conducted using Spearman correlation analysis. We further conducted in *vitro* experiments that the expression of SORBS2 were analyzed, and SORBS2 siRNA were transfected into oxidized LDL (OxLDL)-induced macrophages, followed by western blot and immunofluorescence.

**Results:**

Circulating SORBS2 levels were positively associated with inflammatory factors and lipid indexes. We also observed that high in vitro expression of SORBS2 in OxLDL-induced macrophages. After SORBS2 silencing, Nod like receptor family pyrin domain-containing 3 protein(NLRP3)-Caspase1 activation and NF-κB activation were attenuated, and secretion of pro-inflammatory cytokines (IL-1β and IL-18) was decreased. Moreover, SORBS2 silencing blocked reactive oxygen species (ROS) production and lipid accumulation, and promoted cholesterol efflux through ABCG1-PPARγ pathway.

**Conclusions:**

SORBS2 regulates lipid-induced inflammation and foam cell formation, and is a potential therapeutic target for hypercholesterolemia.

**Supplementary Information:**

The online version contains supplementary material available at 10.1186/s12967-022-03381-z.

## Introduction

Atherosclerosis (AS) is a lipid-induced chronic inflammatory disease characterized by arterial lesions, plaque accumulation, and ultimately plaque rupture. Patients with familial hypercholesterolemia (FH), a disease characterized by premature atherosclerosis, are especially prone to present atherosclerosis-related adverse clinical events. Within the atherosclerosis lesions, monocyte-derived macrophages are the most abundant subset of innate immunity cells, and these macrophages are known to play a critical role in lipid accumulation and inflammatory response [1]. To effectively protect against atherosclerosis-related adverse clinical events, interventions in atherosclerosis (especially in FH patients) should target these initial inflammatory processes.

Sorbin and SH3 Domain Containing 2 (SORBS2) is a key member of the sorbin homology family of adapter and scaffold proteins. Recent studies suggest that SORBS2 is a potential biomarker for cardiac disease. Li et al*.* reported the potential role of SORBS2 in the development of heart failure in left ventricular noncompaction cardiomyopathy [2]. In addition, Kakimoto et al*.* reported that SORBS2 is released from damaged cardiac tissue into the bloodstream upon lethal acute myocardial infarction [3]. Elevated expression of serum SORBS2 in patients with FH was also indicated through bioinformatic analysis. However, the role of SORBS2 in hypercholesterolemia and its underlying molecular mechanisms remain poorly understood.

As noted above, inflammation plays a pivotal role in atherosclerosis. The Nod like receptor (NLR) family pyrin domain-containing 3 protein (NLRP3) inflammasome, is inducibly expressed in macrophages (and other cells) in response to inflammatory stimuli. NLRP3 is known to be activated by a range of injury associated signals, including reactive oxygen species (ROS), oxidized LDL (OxLDL) and cholesterol crystals [4–6]. In a diet-induced atherosclerosis mouse model, NLRP3 inflammasome activation was linked to atherosclerosis progression via gene silencing of *Nlrp3* in *Apoe*^−/−^ mice [7]. An increase in NLRP3 expression has also been demonstrated in human carotid AS plaque tissue compared to non-AS iliac arteries [8]. Thus, interfering with the NLRP3 inflammasome pathway may be a useful strategy to reduce local inflammation and inhibit plaque progression. While NLRP3 silencing may reduce local inflammation, it remains to be proven that SORBS2 modulates OxLDL-induced foam cell inflammation via the NLRP3 inflammasome pathway.

Macrophage foam cell formation is a typical pathological change associated with early atherosclerosis. PPARs play an essential role in macrophage foam cell formation by affecting lipid uptake and efflux. PPARγ is ligand-inducible transcription factor that is highly expressed in macrophages, controlling macrophage inflammation, polarization, and lipid metabolism in atherosclerosis plaques. However, the potential role of SORBS2 in lipid metabolism has not yet to be fully explored.

To address these questions, we examined the correlation between SORBS2 and inflammatory factors, investigated the effects of SORBS2 on inflammation and lipid metabolism by silencing SORBS2, and elucidated the molecular mechanism underlying SORBS2 modulation of OxLDL-induced macrophages.

## Materials and methods

### Population study and experimental design

The present study included 30 patients with a genetic diagnosis of heterozygous FH, and thus lifelong exposure to high LDL cholesterol levels, and 30 non-FH control participants (all relatives of the FH patients). All the FH patients were had a molecular and/or clinical DLCN score ≥ 6 according to guidelines. Demographic, lifestyle, general medical, lipid-lowering treatment, and therapeutic data were obtained for all participants. While all patients with FH were receiving lipid-lowering treatment according to clinical guidelines, none had yet reached LDL target levels. Thus, the mean level of LDL cholesterol in the FH group was 7.76 mmol/L, and the mean level of LDL cholesterol in controls was 3.40 mmol/L (Table 1). Neither the FH group nor the control group included patients with decompensated heart failure, severe hepatic or renal insufficiency, thyroid dysfunction, systemic inflammation, or malignant tumors. Blood samples from patients and healthy controls were collected for ELISA analysis as described below.

**Table 1:** Clinical characteristics of patients with familial hypercholesterolemia and their relatives

Characteristic	Patients with FH	Relatives without FH	*p* value
No	30	30	
Age, mean ± SD, y	45.0 ± 15.8	42.5 ± 16.5	0.588
Male, No. (%)	18 (60.0%)	16 (53.3%)	0.602
BMI, mean ± SD	23.3 ± 3.4	24.7 ± 2.8	0.836
Hypertension	10 (33.3%)	12 (40.0%)	0.592
Diabetes mellitus	3 (10.0%)	5 (16.7%)	0.448
CAD	16 (53.3%)	–	–
Current smoking	8 (26.7%)	7 (23.3%)	0.766
Lipid-lowering therapy	28 (93.3%)	–	–
TC, mmol/L	9.23 ± 2.17	5.35 ± 3.15	** < 0.001**
LDL-C, mmol/L	7.76 ± 1.09	3.40 ± 2.71	** < 0.001**
TG, mmol/L	1.79 ± 1.53	1.63 ± 1.00	0.655
HDL-C, mmol/L	1.25 ± 1.10	1.34 ± 0.42	0.673

*FH* familial hypercholesterolemia, *BM*I body mass index (calculated as weight in kilograms divided by height in meters squared), *CAD* coronary artery disease, *TC* total cholesterol, *LDL-C* low density lipoprotein cholesterol, *TG* triglyceride, *HDL-C* high density lipoprotein cholesterol

This study complied with the Declaration of Helsinki and was approved by the hospital’s ethical review board (FuWai Hospital & National Center for Cardiovascular Diseases, Beijing, China). Each participant provided written, informed consent before enrolment.

### Bioinformatics analysis

We performed differential gene expression analysis of the GSE6054 (10 FH monocyte and 13 control participants) and GSE6088 (10 FH T cells and 13 control participants) microarray datasets in the GEO database (http://www.ncbi.mlm.nih.gov/geo/). The Linear Models for Microarray (LIMMA; Version: 3.30.3) in R package was used to screen out differentially expressed genes (DEGs) from FH patients and of matched controls in these expression profile datasets. This was achieved using an adjusted *P* value < 0.05 and (|log2FC|> 1) as threshold values. The output of our comparative analysis was a list of significantly up-regulated (log2FC > 1) and down-regulated (log2FC < -1) genes.

### Cell culture and treatment

The human monocyte leukemia cell line (THP-1) was obtained from the Cell Bank of the Type Culture Collection of the Chinese Academy of Sciences. The cells were cultured at a density of 5 × 10^5^ cells/mL in Roswell Park Memorial Institute (RPMI) 1640 medium (Gibco, Carlsbad, CA) supplemented with 10% fetal bovine serum (FBS, Gibco, GrandIsland, NY) at 37℃ in a 5% CO_2_ incubator. In all experiments, THP-1 monocytes were cultured in 6-well plates and treated with 100 nM phorbol 12-myristate 13-acetate (PMA) for 48 h to effect transformation into adherent macrophages. The differentiated macrophages were then treated with 50 μg/mL Ox-LDL for 24 h to induce form cells. The culture supernatants were collected in 1.5 mL tubes for ELISA. For protein extraction, the plates were first washed three times with PBS.

### ELISA for cytokine measurements

To evaluate the concentrations of secreted SORBS2 and cytokines, including NLRP3, caspase-1, IL-1β and IL-18, samples were isolated from human serum and cell culture supernatant and analyzed using ELISA kits from R&D Systems (Minneapolis, USA), Abcam (Cambridge, UK) and Sorlarbio (Beijing, China). All procedures were performed according to instructions from the manufacturers. Each sample was analyzed in duplicate.

### Oil red O staining

THP-1 derived macrophages or foam cells were fixed with 4% paraformaldehyde solution for 30 min and then washed with PBS. All cells were stained with Oil Red O in isopropanol for 3 min and then counterstained with hematoxylin for 20 s. Cell morphology was observed using a Leica microscope system (Leica, Wetzlar, Germany).The numbers and individual sizes of lipid droplets in the images, were analyzed using Image J software (https://imagej.nih.gov/ij/), and are reported as surface area in square micrometers (μm^2^). Data were then loaded into GraphPad Prism 8 to calculate the percentage of Oil Red O-stained area.

### Small interfering RNA transfection

Gene knockdown experiments were performed in serum-free Opti-MEM (Invitrogen, U.S.) using Lipotectamine RNAiMAX Transfection Reagent (Invitrogen, U.S.). Macrophages were transfected for 24 h with 40 nM human SORBS2 small interfering RNA (si-SORBS2) or mock transfected for 24 h with a negative control (si-NC). The human si-SORBS2 sequences (sense, 5′- GCAUCUUCCCUGUUUCCUATT-3′; antisense, 5′-UAGGAAACAGGGAAGAUGCTT-3′) and si-NC sequences (sense, 5′-UUCUCCGAACGUGUCACGUTT-3′; antisense, 5′-ACGUGACACGUUCGGAGAATT-3′) were synthesized by Gene Pharma (Gene Pharma Co., Ltd., China). For the transfection protocol, we prepared two sepatate microcentrifuge tubes, one containing Si-SORBS2 in Opti-MEM and the other containing Si-NC in Opti-MEM. In addition, 2 μL of Lipotectamine RNAiMAX reagent was separately mixed with 100 μL Opti-MEM, and the tube was incubated for 5 min. Each diluted RNAi duplex was then mixed with diluted Lipofectamine (51 μL). Both mixtures were then incubated for 20 min at room temperature. Next, the two mixtures were separately added to macrophages cultured in 6-well plates, and the plates were incubated for 24 h at 37 ℃. Finally, the macrophages were treated with 50 μg/mL OxLDL for 24 h to induce foam cells. These cells were subsequently harvested for western blot analysis.

### Measurement of cellular reactive oxygen species

ROS production was evaluated using a Reactive Oxygen Species (ROS) Detection Assay Kit. Seed 2.5 × 10^4^ THP-1 derived macrophages per well in 96-well plate to obtain 70–80% confluency. Then adherent cells were transfected with Si-SORBS2 or Si-NC as described above. After cells treated with or without OxLDL overnight, a fluorescent probe, 2‵7‵-dichlorofluorescin diacetate (DCFDA), was directly added into serum-free medium (final concentration 10 μM) and incubated for 30 min at 37 ℃ in the dark. After incubation, the medium was discarded, and the probes were washed with PBS. Upon the cell entry, DCFDA is modified by cellular esterases to form a non-fluorescent DCF. Oxidation of DCF by intracellular ROS yields highly a fluorescent product that can be detected. Measure fluorescence immediately or desired period of time using a fluorescence microplate reader (excitation, 488 nm; emission, 520 nm). For fluorescence microscope analysis, the cells were observed immediately using emission filter appropriate for fluorescein. The fluorescence intensities were measured immediately using a FACS Accuri C6 machine (BD Biosciences) and analyzed using FlowJo V10 (BD Biosciences).

### Cholesterol efflux assays

THP-1 derived macrophages (or foam cells) and conditioned medium were harvested, and intracellular and extracellular cholesterol levels were determined using the Amplex Red Cholesterol Assay Kit (Invitrogen) according to the manufacturer’s instructions. Briefly, macrophages or foam cells were solubilized in cell lysis buffer—nine parts RIPA, one part phenylmethylsulfonyl fluoride (PMSF). The cell extracts were clarified by centrifugation at 12,000*g* for 10 min at 4 ℃, and total protein contents were determined by using a BCA assay kit (Beyotime, Nanjing, China). The cell extracts (μg of protein) and conditioned medium (μg of cholesterol per milliliter of culture medium) were then diluted with 1 × reaction buffer. The extracted cholesterol diluted in reaction buffer were then added to individual wells (50 μL per well) of a 96-well plate. Nest, 50 μL of working reagent containing Amplex Red, cholesterol oxidase, cholesterol esterase, and horseradish peroxidase was added to each well. After 30 min incubation at 37 ℃ in the dark, fluorescence readings (excitation, 550 nm; emission, 590 nm) were taken from each sample. A standard reference curve for cholesterol (0–8 μg/ml) was used to estimate sample cholesterol levels. Cholesteryl ester (CE) content was measured after subtracting (free cholesterol) FC from (total cholesterol) TC. The results are expressed as μg of protein and μg of cholesterol per milliliter of culture medium, respectively.

### Protein extraction and western blotting

Western blotting was performed using standard protocols. Briefly, total cellular protein was extracted using lysis buffer (Beyotime, Beijing, China) supplemented with protease, phosphatase inhibitor cocktail (Beyotime, Beijing, China), and PMSF (Beyotime, Beijing, China). To extract nuclear fractions, the Nuclear Protein Extraction Kit was used according to the manufacturer’s instructions (Solarbio, Beijing, China). All extracted proteins were stored at 80 ℃. Sample protein concentrations were determined using the bicinchoninic acid method. Prior to analysis by SDS-PAGE, protein samples were denatured at 100 °C for 5 min. The protein samples were then resolved by electrophoresis and transferred to NC membranes (Invitrogen, USA). After transfer, the membranes were blocked with 5% nonfat milk at room temperature for 2 h. The membranes were then incubated with specific primary antibodies (see below) overnight at 4 ℃. The primary antibodies against NLRP3, Caspase 1, cleaved caspase 1, ASC, IL-1β, cleaved IL-1β, NF-κB p65, phosphor-NF-κB p65 (Ser 536), ABCA1, PPARγ, and GAPDH were purchased from Cell Signaling Technology (Boston, USA). The antibody against ABCG1 was purchased from Abcam (Cambridge, UK), and the antibody against SORBS2 was purchased from Proteintech (Chicago, USA). After washing, the membranes were incubated with appropriate secondary antibodies for 1.5 h at room temperature. Finally, bound antibody bands were visualized using a GE ImageQuantLAS4000 Chemiluminescent instrument (GE, USA). All the western blotting images presented in this study are representative of at least three independent biological experiments. The band densities were quantified by TotalLab image analysis software after normalizing to GAPDH.

### Immunofluorescence staining

THP-1 derived macrophages or foam cells were fixed with 4% paraformaldehyde for 20 min, and then permeabilized with 0.3% TritonX-100 for 2 min. After antigen retrieval and blocking, the slides were incubated with primary antibody at 4 ℃ overnight. After discarding the primary antibody, the slides were incubated with Alexa Fluor® 488 goat anti-rabbit IgG (H + L) antibody for 1 h at 37 °C. The nuclei were counterstained with 4′,6-diamidine-2-phenylindole (DAPI; Beyotime, China) before observation. Images were captured using an Olympus FV1000 confocal microscope (Olympus, Japan) at 100× magnification.

### Statistical analysis

All experiments were performed independently at least three times. Continuous variables are reported as mean ± standard deviation (SD) or median [interquartile range (IQR)]. Categorical variables are reported as number (percentage). The between group characteristics of participants were analyzed using Student *t* test and ANOVA tests. For the correlation analyses between SORBS2 and inflammatory cytokines, we calculated Spearman correlation coefficients (r). Analyses were performed using IBM SPSS Statistics version 25.0 (IBM SPSS Statistics, IBM Corp., Armonk, NY, USA), GraphPad Prism 8.0 software and R (http://www.r-project.org/) statistical packages. Differences with a *P* value < 0.05 were considered statistically significant.

## Results

### SORBS2 expression is up-regulated in patients with familial hypercholesterolemia and in OxLDL-induced THP-1 macrophages

**Fig. 1:**
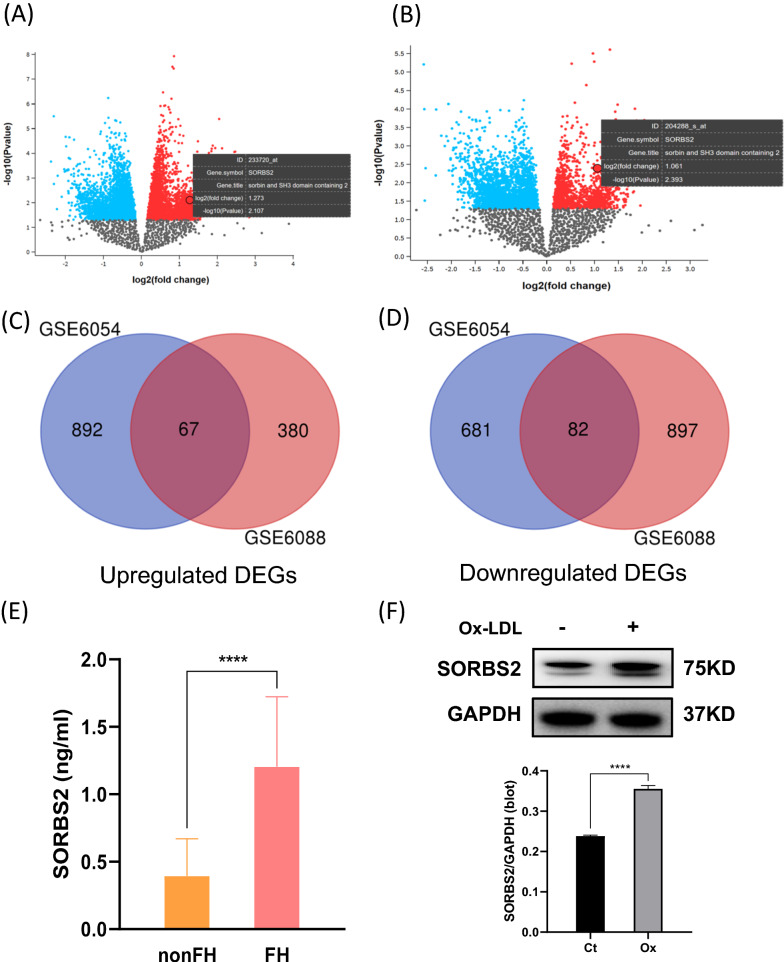
SORBS2 is upregulated in patients with familial hypercholesterolemia. Bioinformatics analysis was used to identify differentially expressed genes (DEGs) in FH patients and normal control participants. **A** Volcano plot of DEGs in FH and control samples in GSE6054. **B** Volcano plot of DEGs in FH and control samples in GSE6088. **C** Venn diagram representing a total of 67 upregulated genes included in the two datasets. **D** Venn diagram representing a total of 82 downregulated genes included in the two datasets. **E** ELISA results showing concentrations of SORBS2 in serum from FH patients and normal control participants (n = 30). **F** THP-1 derived macrophages treated with or without Ox-LDL (50 μg/mL) for 24 h. The protein levels were determined by western analyses. **** *P* < 0.0001 compared with the control group (no Ox-LDL treatment). DEGs, differentially expressed genes; FH, familial hypercholesterolemia

We first conducted a bioinformatics analysis of GEO datasets to identify differentially expressed genes in patients with FH compared with control participants. Using the GEO2R online tool, we identified 1722 DEGs in GSE6054 (959 upregulated, 763 downregulated), and 1426 DEGs in GSE6088 (447 upregulated, 979 downregulated), that were differentially expressed between FH patients and control participants (see volcano plots in Fig. 1A, B) A Venn diagram showing the overlap between differentially upregulated genes in the two datasets is shown in Fig. 1C, and a Venn diagram showing the overlap between differentially downregulated genes in the two datasets is shown in Fig. 1D. The results reveal that there were 149 overlapping DEGs (67 upregulated, 82 downregulated) in the two datasets. Of the top 20 upregulated genes, SORBS2 was found to be associated with coronary artery disease, and was chosen as a gene of interest for further study (Additional file 1: Table S2). To validate the results of our DEG analyses, we performed an ELISA on FH patients control participant serum samples. The ELISA results demonstrate a significant increase in the concentration of SORBS2 in patients with FH compared with controls (1.20 ng/mL vs. 0.39 ng/mL, *P* < 0.0001; Fig. 1E). Thus, the ELISA results confirm the results of our DEG analyses. We further used THP-1 derived macrophages treated with OxLDL (50 μg/mL) to mimic hypercholesterolemia in vitro. The western blot result showed that SORBS2 was upregulated in the OxLDL-induced macrophages, which is similar to the results that SORBS2 levels are upregulated in FH patients (Fig. 1F).

### Circulating SORBS2 is correlated with inflammation factors and lipid indexes

**Fig. 2:**
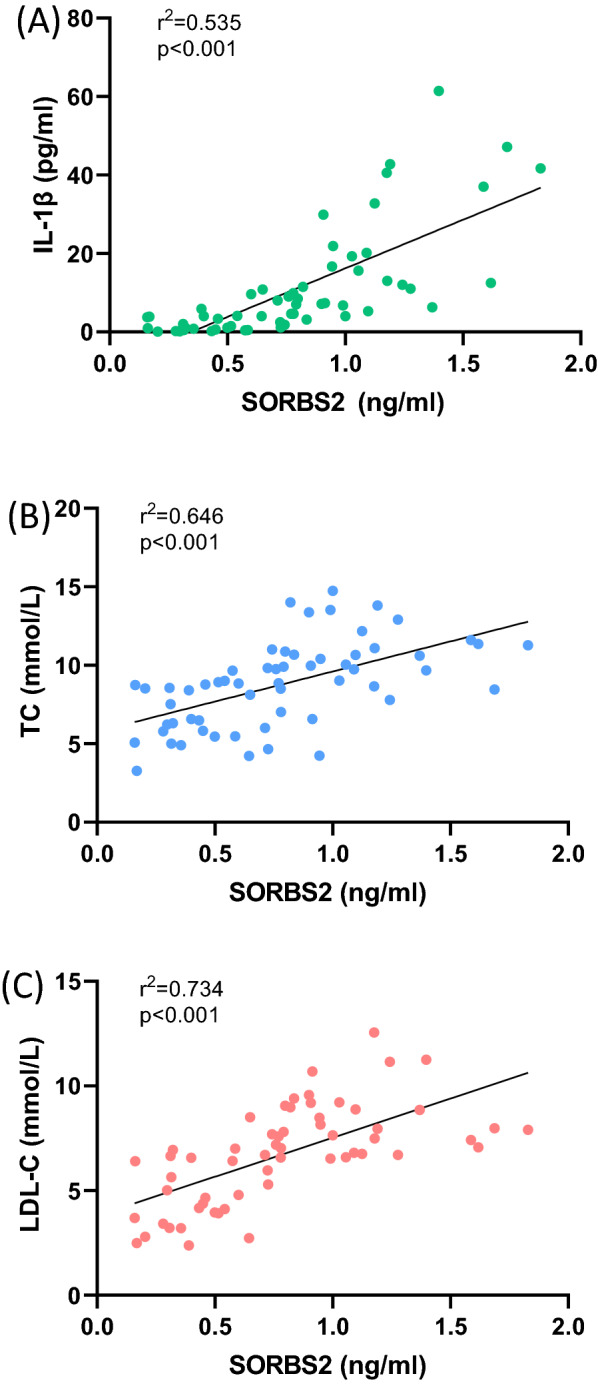
SORBS2 is correlated with several inflammatory factors and lipid indexes levels in human serum. Spearman correlation analyses demonstrated that SORBS2 levels were positively associated with several inflammatory factors. **A** IL-1β; **B** TC; and **C** LDL-C

The baseline characteristics of FH patients and control participants are shown in Table 1. In addition to circulating SORBS2 levels, we compared the levels of inflammatory factors and lipid indexes between patients in the FH group and control participants (Additional file 1: Fig. S1). Interestingly, we found a moderate but significant correlation between circulating SORBS2 levels and the secretion of IL-1β (Spearman r = 0.731, *P* < 0.001). Furthermore, SORBS2 levels were also positively correlated with the levels of TC (Spearman r = 0.804; *P* < 0.001), and LDL-C (Spearman r = 0.857; *P* < 0.001), indicating that circulating SORBS2 levels were positively associated with lipid indexes (Fig. 2). However, no association between SORBS2 levels and TG levels or between SORBS2 levels and HDL-C levels was observed (Additional file 1: Fig. S1).

### SORBS2 silencing attenuates OxLDL-induced inflammation through the NLRP3 inflammasome and NF-κB signaling pathways

**Fig. 3:**
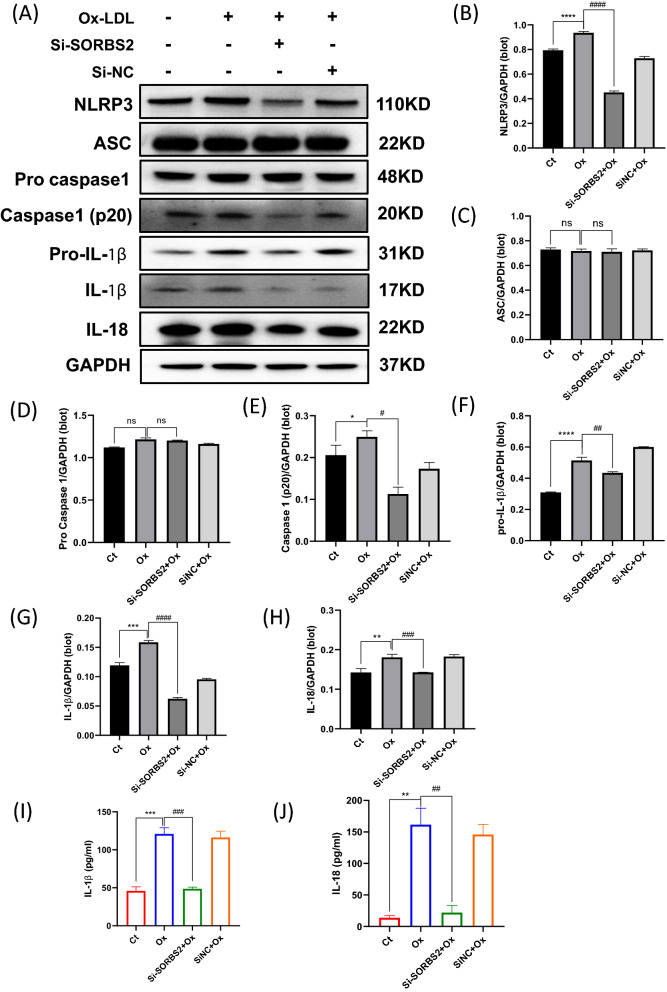
SORBS2 siRNA attenuates activation of the NLRP3 inflammasome. **A–H** THP-1 cells were stimulated with OxLDL (50 μg/L and treated with or without Si-SORBS2 for 24 h. Protein expression levels were then measured by western blotting analysis. **B** NLRP3; **C** ASC; **D** pro-caspase-1; **E** caspase-1 p20; **F** pro-IL-1β; **G** IL-1β; **H** IL-18. GAPDH levels were used as an internal control. The histogram reports mean ± SEM of protein band density from three experiments (normalized by comparison with GAPDH). **I–J** ELISA results showing inflammatory cytokine concentrations in the culture media. **I** IL-1β; **J** IL-18. * *P* < 0.05, ** *P* < 0.01, and *** *P* < 0.001 compared with the control group (no OxLDL treatment); ^#^*P* < 0.05, ^##^ *P* < 0.01, ^###^ *P* < 0.001 compared with the corresponding controls (OxLDL at 50 μg/mL)

**Fig. 4:**
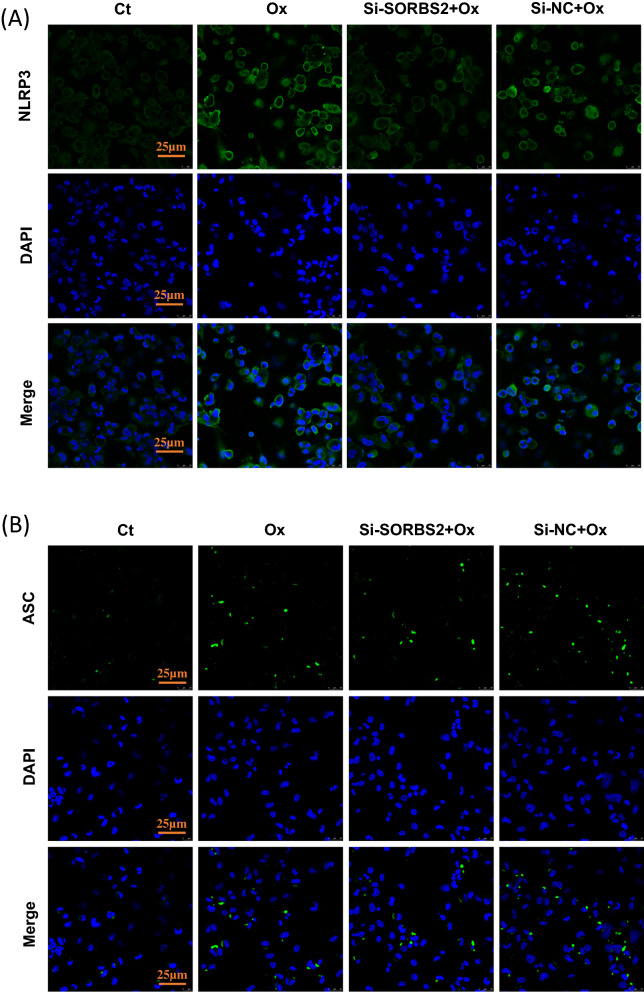
NLRP3 expression and ASC speck formation in THP-1 derived macrophages. **A** NLRP3 expression was visualized by immunofluorescence analysis with an anti-NLRP3 (green) antibody in THP-1 macrophages pretreated with or without Si-SORBS2 followed by Ox-LDL (50 µg/mL) for 24 h. DAPI (blue) was used as a nuclear marker. Scale bar, 25 µm. **B** ASC speck formation was visualized by immunofluorescence analysis with an anti-ASC (green) antibody in THP-1 macrophages pretreated with or without Si-SORBS2 followed by OxLDL (50 µg/mL) for 24 h. DAPI (blue) was used as a nuclear marker. Scale bar, 25 μm (400 ×)

Activation of the NLRP3 inflammasome has previously been demonstrated to play an important role in the severe inflammation underlying atherosclerosis [6]. In the present study, OxLDL was used to trigger activation of the NLRP3 inflammasome in macrophages [9]. These activated macrophages were also transfected with SORBS2 siRNA (or a negative control) and to investigate the effects of si-SORBS2 silencing on NLRP3 inflammasome activation. While OxLDL activation increased the expression of NLRP3 protein, this effect was reversed by si-SORBS2 treatment (Fig. 3A, B). Thus, si-SORBS2 silencing induced an anti-inflammatory effect by reducing NLRP3 inflammasome activation. The effect of si-SORBS2 on NLRP3 inflammasome activation was also confirmed using immunofluorescence staining (Fig. 4A). Moreover, western analyses revealed that si-SORBS2 blocked an associated increase in cleaved caspase-1(p20) (Fig. 3A, D, E), but did not change ASC protein expression (Fig. 3A, C) Interestingly, si-SORBS2 also reduced the number of ASC specks stimulated by OxLDL (Fig. 4B). Together, these observations provide evidence that SORBS2 silencing can inhibit activation of the NLRP3 inflammasome pathway. In addition, SORBS2 silencing was observed to suppress an OxLDL-induced increase in IL-1β and IL-18 inflammatory factor levels in cell lysate (Fig. 3F–H) and supernatant (Fig. 3I, J).

**Fig. 5:**
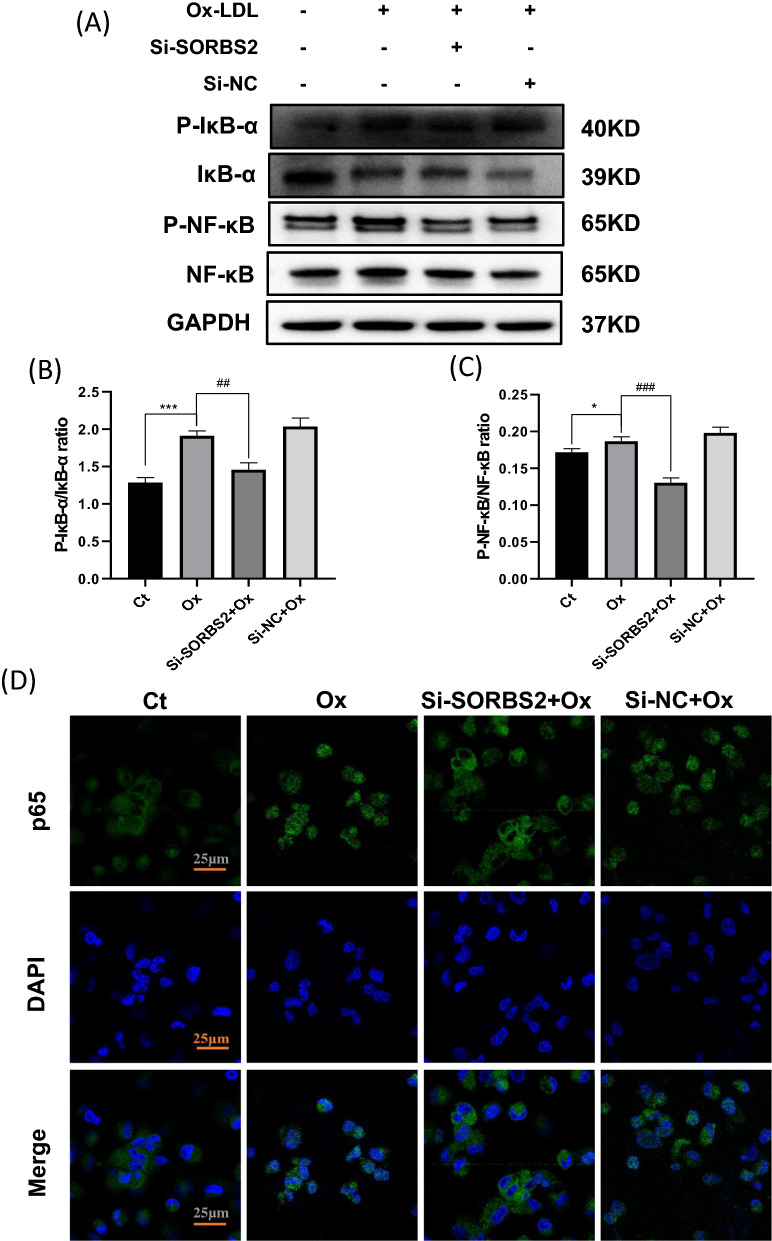
SORBS2 silencing inhibits activation of NF-κB. **A–C** Si-SORBS2 treatment inhibited NF-κB signaling in THP1-derived macrophages. Western blotting was performed to evaluate p-IκB-α, IκB-α, p-p65, p65, and GAPDH protein expression levels in macrophages treated with or without Si-SORBS2. **D** p65 subunit protein levels in the cytoplasm and nucleus were also detected by confocal microscopy. p65 nuclear localization was visualized with an anti-p65 (green) antibody in THP-1 derived macrophages pretreated with Si-SORBS2 followed by OxLDL for 24 h. DAPI (blue) was used as a nuclear marker. The histogram reports mean ± SEM of densitometric scans of protein bands from three experiments (normalized by comparison with GAPDH). * *P* < 0.05, ** *P* < 0.01, and *** *P* < 0.001 compared with the control group (no Ox-LDL treatment); ^#^*P* < 0.05, ^##^*P* < 0.01, ^###^*P* < 0.001 compared with the corresponding controls (OxLDL at 50 µg/mL). Scale bar, 25 μm (400 ×)

Nuclear factor κB (NF-κB) is a key transcriptional factor playing an important role in inflammation by inducing upregulation of various inflammatory genes, and hence it is involved in inflammation [10]. Macrophages were treated with OxLDL (50 μg/mL) for 15 min, 30 min, 1 h, 2 h, 3 h, 6 h, 12 h, 24 h. Then we collected the proteins at different time points and did western blot analysis of phosphorylated NF-κB. Finally, we found OxLDL was demonstrated to increase phosphorylation of NF-κB in a time-dependent manner, peaking at 3 h (Additional file 1: Fig. S2A, B). However, SORBS2 silencing suppressed the expressions of p-p65 and p-IκB at 3 h after treatment (Fig. 5A). Moreover, the p-p65/p65 and p-IκB/IκB ratios were reduced in OxLDL-induced cells expressing si-SORBS2 (Fig. 5B, C). In addition, the immunofluorescence staining revealed that si-SORBS2 transfection significantly inhibited nuclear localization of p65, which is consistent with inhibition of NF-κB activity (Fig. 5D).

### OxLDL-induced ROS production is decreased by SORBS2 silencing

**Fig. 6:**
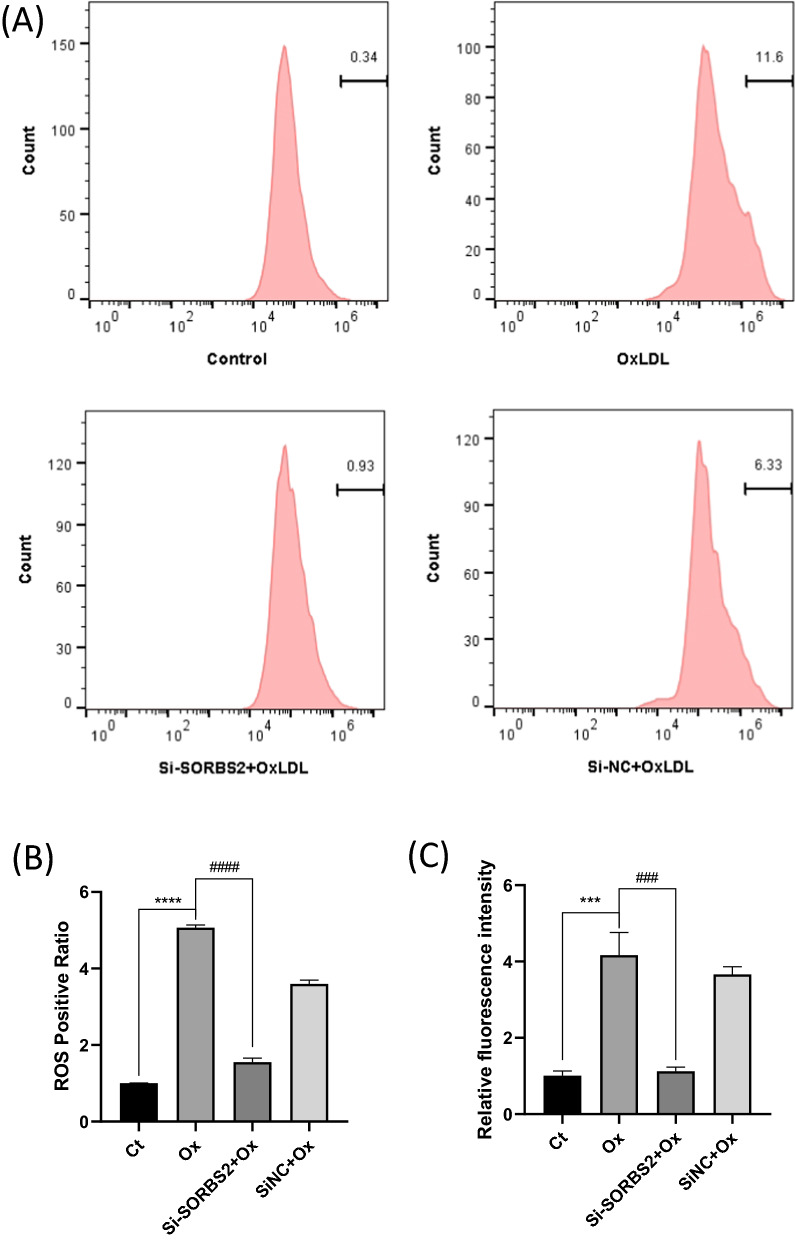
SORBS2 siRNA inhibits ROS production. THP-1 derived macrophages were pretreated with Si-SORBS2 or Si-NC for 24 h and then stimulated with OxLDL (50 µg/mL) for 24 h. **A** ROS production was detected by flow cytometry. **B** Densitometric analysis was used to quantify the levels of ROS production (n = 3). **C** Relative fluorescence intensity was also measured using a fluorescence microplate reader (n = 5). *** *P* < 0.001 and **** *P* < 0.0001 compared with the control group (no OxLDL treatment); ^###^*P* < 0.001 and ^####^*P* < 0.0001 compared with the corresponding controls (OxLDL at 50 µg/mL)

Activation of the NLRP3 inflammasome requires a second activation signal from ROS production [11]. In OxLDL-induced macrophages, si-SORBS2 transfection was observed to downregulate intracellular ROS production (Fig. 6). Thus, SORBS2 silencing impairs the second signal required for NLRP3 activation.

### SORBS2 silencing on oxLDL-induced lipid accumulation in THP-1 macrophages

**Fig. 7:**
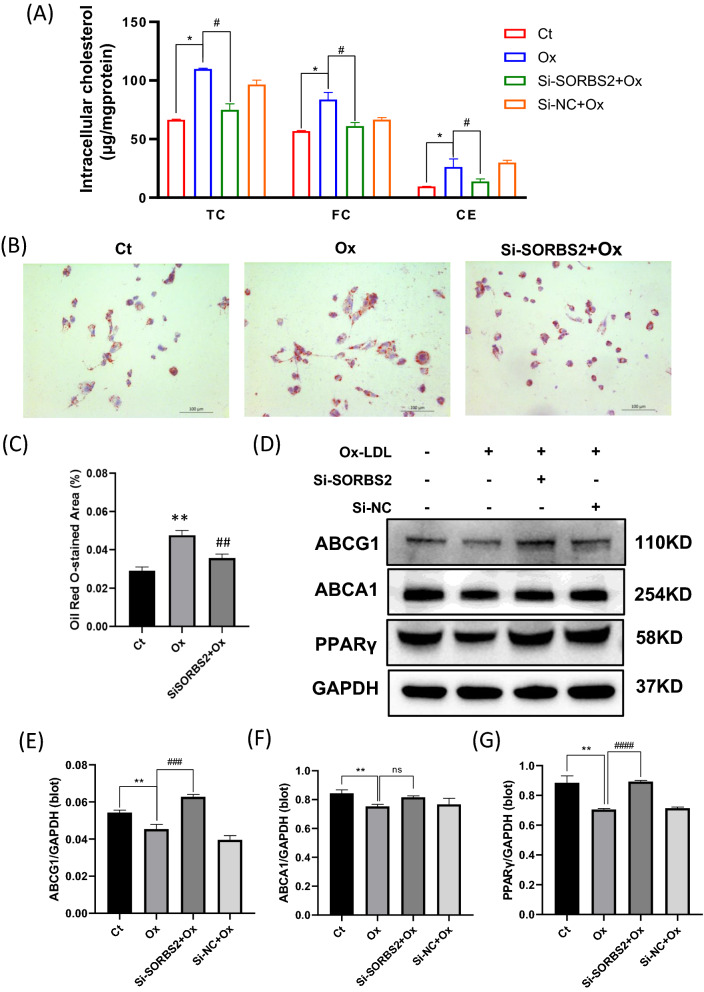
Effects of SORBS2 silencing on lipid accumulation and cholesterol efflux in OxLDL-induced THP-1 macrophages. **A** Total cholesterol (TC), free cholesterol (FC), and cholesterol ester (CE) levels were measured in culture supernatant from OxLDL-activated macrophages treated with Si-NC or Si-SORBS2 (n = 5) using the Amplex Red Cholesterol Assay Kit. **B** Representative images of Oil Red O-stained differentiated THP1 cells in each cell group. **C** Quantitative analysis of Oil Red O-stained results. **D–G** Western blotting was performed to quantify ABCA1, ABCG1, and PPARγ protein expression levels in THP-1 derived macrophages. The histogram reports the mean ± SEM of the densitometric scans for the protein bands from three experiments (normalized by comparison with GAPDH). * *P* < 0.05, ** *P* < 0.01, and *** *P* < 0.001 compared with the control group (no Ox-LDL treatment); # *P* < 0.05, ^##^ *P* < 0.01, ^###^*P* < 0.001 compared with the corresponding controls (OxLDL at 50 µg/mL)

The association between aberrant lipid metabolism and inflammation is known to be strong. Our analyses of lipid indexes revealed that SORBS2 silencing significantly lowered TC, FC, and CE levels in conditioned supernatant from OxLDL-induced macrophages (Fig. 7A). Furthermore, Oil Red O staining of OxLDL-induced macrophages confirmed an accompanying decrease in lipid droplet surface area after si-SORBS2 transfection (Fig. 6B, C). According to western analyses, SORBS2 silencing significantly increased ABCG1 expression, which is the transporter protein responsible for cholesterol efflux. In contrast, SORBS2 silencing did not modulate ABCA1 expression. Expression of PPARγ, a key regulator of reverse cholesterol transport, was also upregulated following si-SORBS2 transfection (Fig. 6D–G). Together, our results provide evidence that SORBS2 silencing can inhibit lipid accumulation and promote cholesterol efflux via the ABCG1-PPARγ pathway.

## Discussion

Patients with FH exhibit increased rates of atherosclerosis and high risks of myocardial infarction at a young age. These outcomes are mainly related to lifelong exposure to high plasma cholesterol levels. Multidrug treatment is often required to achieve adequate LDL-C levels. High-intensity statins, ezetimibe, and bile acid sequestrants can be useful therapies. Monoclonal antibodies targeting against PCSK9 act to increase available LDL-R and markedly reduce plasma LDL-C levels. Apart from LDL-C levels, inflammation also act as a critical role for the maintenance of the atherosclerotic process. Recent studies support the view that patients with FH, despite long-term cholesterol-lowering treatments, exhibit enhanced inflammation [12]. Indeed, hypercholesterolemia and inflammation may be considered as “two sides of the same coin” [13]. Although PCSK9 inhibitor is associated with significant reduction of CV risk, recent meta-analysis on RCTs found that short-term PCSK9 inhibitory treatment did not reduce hs-CRP concentrations [14]. Our study may be a useful complement to prescription of patients with FH, since inhibition of SORBS2 could block the inflammation process thus reducing atherosclerosis.

In the present study, circulating SORBS2 levels were observed to be higher in FH patients than in control participants. In particular, we observed positive correlations between circulating SORBS2 levels and inflammatory factors (IL-1β) and between circulating SORBS2 levels and lipid indexes (TC and LDL-C), indicating that SORBS2 may play an important role in inflammation in patients with familial hypercholesterolemia. Based on these observations, we treated THP1-derived macrophages with OxLDL to mimic hypercholesterolemia, and then investigated whether SORBS2 could regulate inflammatory process and the formation of macrophage-derived foam cells, and thus contribute to the progression of atherosclerosis (AS) in hypercholesterolemic populations.

SORBS2 is a key factor bridging inflammatory dysfunction and atherosclerotic coronary artery disease. SORBS2 is the archetypal member of a three-protein family that includes CAP (SORBS1) and vinexin (SORBS3) [15]. The important role played by SORBS2 in various diseases has previously been reported [2, 15]. SORBS2 variants are also known to be associated with CHD [16]. Moreover, SORBS2 in human heart tissue, where it is localized in the Z-bands of mature myofibrils, has been reported to regulate important processes in cardiomyocytes [3, 17]. In the present study, we report that SORBS2 expression is a marker for the progression of AS. Thus, circulating SORBS2 levels are significantly higher in patients with FH, and patients with FH have higher levels of inflammasome-related cytokines Wang et al. previously reported that SORBS2 can modulate the levels of IL-6, TNF-α, and IL-1β in sepsis-associated cardiac dysfunction [18]. Here, we found that SORBS2 silencing inhibited the secretion of IL-1β and IL-18 into culture supernatant after OxLDL induction of macrophage activation. Moreover, SORBS2 silencing suppressed IL-1β and IL-18 levels in cell lysate. Hence, SORBS2 silencing inhibits inflammation in OxLDL-induced foam cells.

The NLRP3 inflammasome, the best characterized pattern recognition receptor in innate immune response, is comprised of a sensor (NLRP3 protein), an adaptor (ASC protein), and an effector (pro-caspase-1). Epidemiological studies provide indirect evidence that patients with AS display high aortic expression of NLRP3, and that NLRP3 expression levels are correlated with disease severity and clinical risk factors [19]. After activation stimulation, NLRP3 protein interacts with apoptosis-associated speck-like protein containing CARD (ASC) and pro-caspase-1 to form the NLRP3 inflammasome. This ultimately results in cleavage and maturation of the potent pro-inflammatory cytokines IL-1β and interleukin 18 (IL-18) by mature caspase-1 [20, 21]. Mechanistically, activation of the NLRP3 inflammasome proceeds through the NF-κB pathway.

OxLDL-induced macrophages significantly promoted the activation of NF-κB. After NF-κB activation, NF-κB is translocated into the nucleus, thus enhancing NLRP3 inflammasome complex formation and activation. SORBS2 silencing effectively abolished both NF-κB activation and NLRP3 inflammasome activation in macrophages. When the NLRP3 inflammasome becomes activated, the adaptor protein ASC assembles into a large protein complex named an ASC speck, and this is considered a typical sign of inflammasome activation. ASC specks assembled in response to inflammasome activation can be visualized under a microscope as micrometer-sized foci. Here, we investigated the assembly of ASC specks in vitro by immunofluorescence. We found that SORBS2 silencing blocked the assembly of ASC specks in Ox-LDL stimulated macrophages.

Following ASC speck assemblys, ASC nucleates precursor caspase-1, and the NLRP3 inflammasome then cleaves precursor caspase-1 into active caspase-1. Subsequently, activated caspase-1 processes IL-1β and IL-18 precursors into mature IL-1β and IL-18, augmenting the inflammatory response and associated impairments. Our studies reveal that SORBS2 silencing inhibited activation of the NLRP3/ caspase-1/ IL-1β pathway, ultimately blocking the inflammation process in AS, and thus potentially providing protection against hypercholesterolemia and atherosclerosis [22].

Activation of the NLRP3 inflammasome is reported to require additional triggers such as reactive oxygen species (ROS). ROS are unstable and highly reactive molecules produced by reduction of oxygen mainly during mitochondrial oxidative phosphorylation [23]. In endothelial cells, Ox-LDL treatment under high glucose conditions increases intracellular ROS production, and this subsequently activates the NLRP3 inflammasome [24]. Application of antioxidants signifies a rational curative strategy to prevent disease involving oxidative stress. Natural antioxidants often possess strong antioxidant and free radical scavenging abilities as well as anti-inflammatory action, which are also supposed to be the basis of other bioactivities and health benefits. Here in our study, we found that SORBS2 could regulate the NLRP3 inflammasome through ROS activation. Hence, SORBS2 silencing could act as a natural antioxidants for patients with familial hypercholesterolemia.Our findings reveal that SORBS2 silencing inhibited OxLDL-induced ROS production. By decreasing the production of oxidative stress markers, SORBS2 silencing further inhibits activation of the NLRP3 inflammasome.

In atherosclerosis, formation of macrophage foam cells is considered to be the initial step in the pathological process [25]. These cells are known to regulate OxLDL uptake and intracellular cholesterol trafficking. OxLDL has been reported to play a causative role in the genesis and progression of AS which acts via binding to scavenger receptors and accumulating in the cytoplasm. Our data reveal that foam cell formation was enhanced in THP-1 cells under OxLDL conditions, as demonstrated by Oil Red O staining. It was previously established that receptor-mediated cholesterol efflux and reverse cholesterol transplant are major mechanisms in the removal of cellular cholesterol, and CE hydrolysis is the rate-limiting step in these processes. In OxLDL treated THP-1 cells, increased cholesterol efflux (TC, FC, and CE) was observed to downregulate ABCG1 and PPARγ. These results are consistent with the Bekkering et al*.* study. [26] Because SORBS2 silencing significantly increased TC, FC, and CE efflux, and it can be concluded that SORBS2 plays a role in Ox-LDL-induced macrophage foam cell formation.

The ligand-inducible transcription factors PPARγ is highly expressed in macrophages, and is known to control macrophage inflammation, polarization, and lipid metabolism in atherosclerosis plaques [27]. Previous studies have demonstrated a crucial role for PPARγ in the induction of ABCA1/ ABCG1 expression, and the prevention of foam cell formation and atherosclerosis progression [28]. Jiang el al. reported that PPARγ ligands markedly reduce induction of ABCG1 expression and HDL-mediated cholesterol efflux [29]. In the present study, We found that SORBS2 silencing augmented ABCG1 protein expression and cholesterol efflux to HDL. In contrast, SORBS2 silencing did not influence ABCA1 protein expression and thus does not affect cholesterol efflux to ApoA1. Therefore, it can be speculated that SORBS2 silencing regulates macrophage foam cell formation by promoting reverse cholesterol transport. Taken together, our results provide further evidence that SORBS2 can regulate lipid biosynthesis through transcriptional activation of lipogenic genes.

## Conclusion

In summary, our study provides evidence that SORBS2 silencing regulates foam cell formation and relieves inflammation by blocking activation of both the NLRP3 inflammasome and NF-κB. In addition, SORBS2 silencing reduces ROS production. Through these mechanisms, SORBS2 silencing prevents cellular lipid accumulation and promotes cholesterol efflux via the PPARγ/ABCG1 pathway. Our findings shed new light on the therapeutic mechanisms of hypercholesterolemia and atherosclerosis.

## Supplementary Information

**Additional file 1:** **Figure S1.** ELISA results showing concentrations of (**A**) IL-1β, (**B**) IL-18, (**C**) IL-8, and (**D**) TNF-α in serum from FH patients and normal control participants (n=30). Spearman correlation analyses demonstrated that SORBS2 levels were not significantly associated with (**E**) HDL-C; and (**F**) TG. **Figure S2.** The expression of NF-κB phosphorylation after stimulation upon ox-LDL at different time using western blotting analysis (n=3) (**A–B**) Macrophages were treated with Ox-LDL (50 μg/mL) for 15min, 30min, 1h, 2h, 3h, 6h, 12h, 24h. Then we collected the proteins at different time points and did western blot analysis of phosphorylated NF-κB. **Figure S3.** Production of ROS were measured using a Reactive Oxygen Species (ROS) Detection Assay Kit. (**A**) The cells were observed using fluorescence microscope. (**B**) Relative fluorescence intensity at different time points were measured using a fluorescence microplate reader (n=3).

**Additional file 2:** **Table S1.** Top 20 upregulated overlapping DEGs in GSE6054 and GSE6088.

**Additional file 3:** Central illustration.

## Data Availability

All data will be shared upon reasonable request to the corresponding author. The datasets used and/or analyzed during the current study are available from the corresponding author on reasonable request.

## Abbreviations

FH: Familial hypercholesterolemia
AS: Atherosclerosis
SORBS2: Sorbin and SH3 Domain Containing 2
CHD: Coronary heart disease
OxLDL: Oxidized low-density lipoprotein
ROS: Reactive oxygen species
NLRP3: Nod like receptor family pyrin domain-containing 3 protein
PPAR-γ: Peroxisome proliferator-activated receptor gamma
DLCN: Dutch lipid clinic network
DEG: Differentially expressed gene
THP-1: Human monocyte leukemia cell line
DCFH-DA: 2′7’-Dichlorofluorescin diacetate
PMA: Phorbol 12-myristate 13-acetate
si-SORBS2: SORBS2 small interfering RNA
CE: Cholesteryl ester
FC: Free cholesterol
TC: Total cholesterol
GAPDH: Glyceraldehyde-3-phosphate dehydrogenase
LDL-C: Low-density lipoprotein cholesterol
ABCA1/G1: ATP-binding cassette transporter A1/G1
