# Supplementary figures and images for: SORBS2 as a molecular target for atherosclerosis in patients with familial hypercholesterolemia
Source: J Transl Med. 2022 May 19;20:233. doi: 10.1186/s12967-022-03381-z (PMC9118763; doi:10.1186/s12967-022-03381-z)

## Slide 1
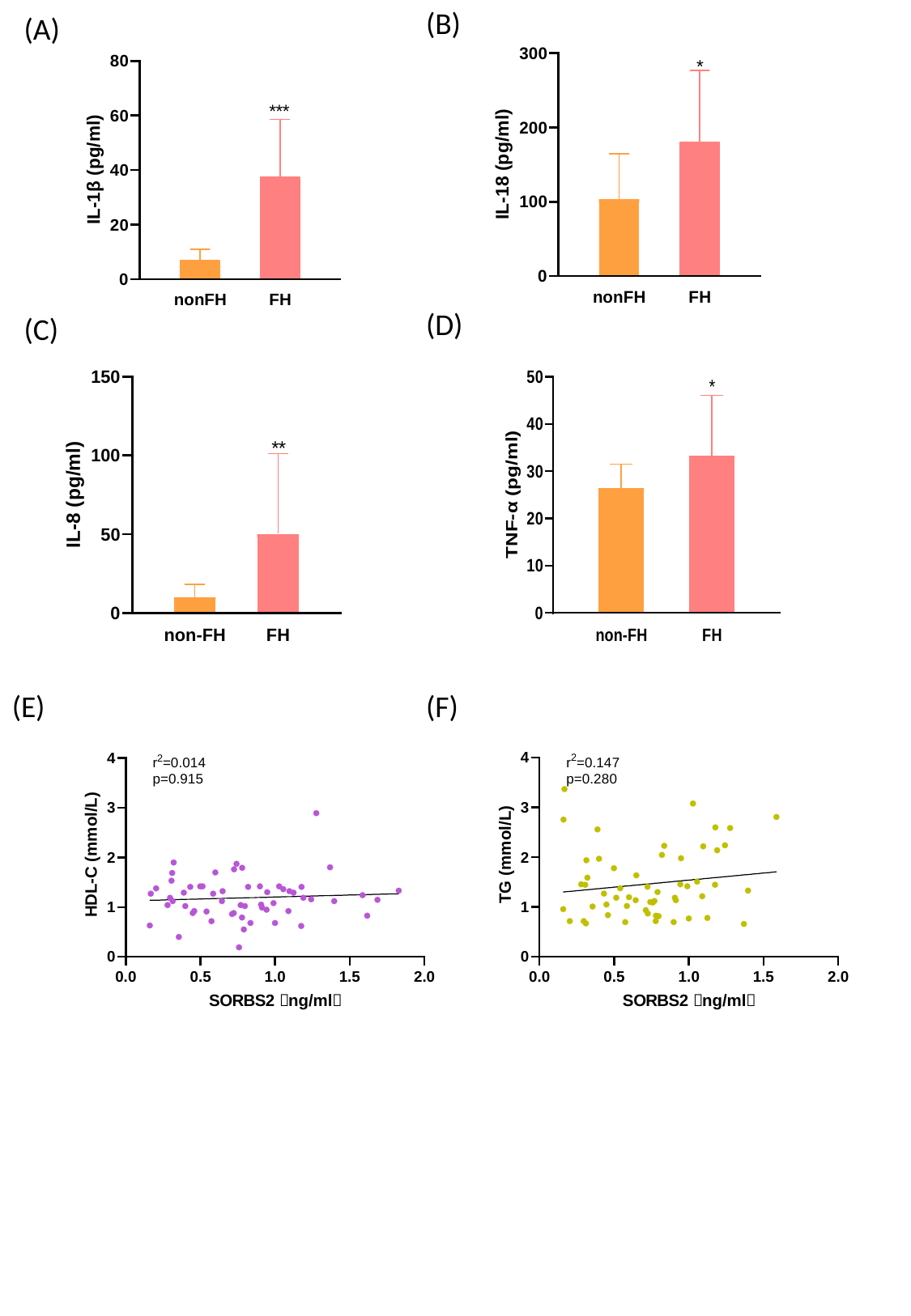

(B)
(A)
(D)
(C)
(E)
(F)

## Slide 2
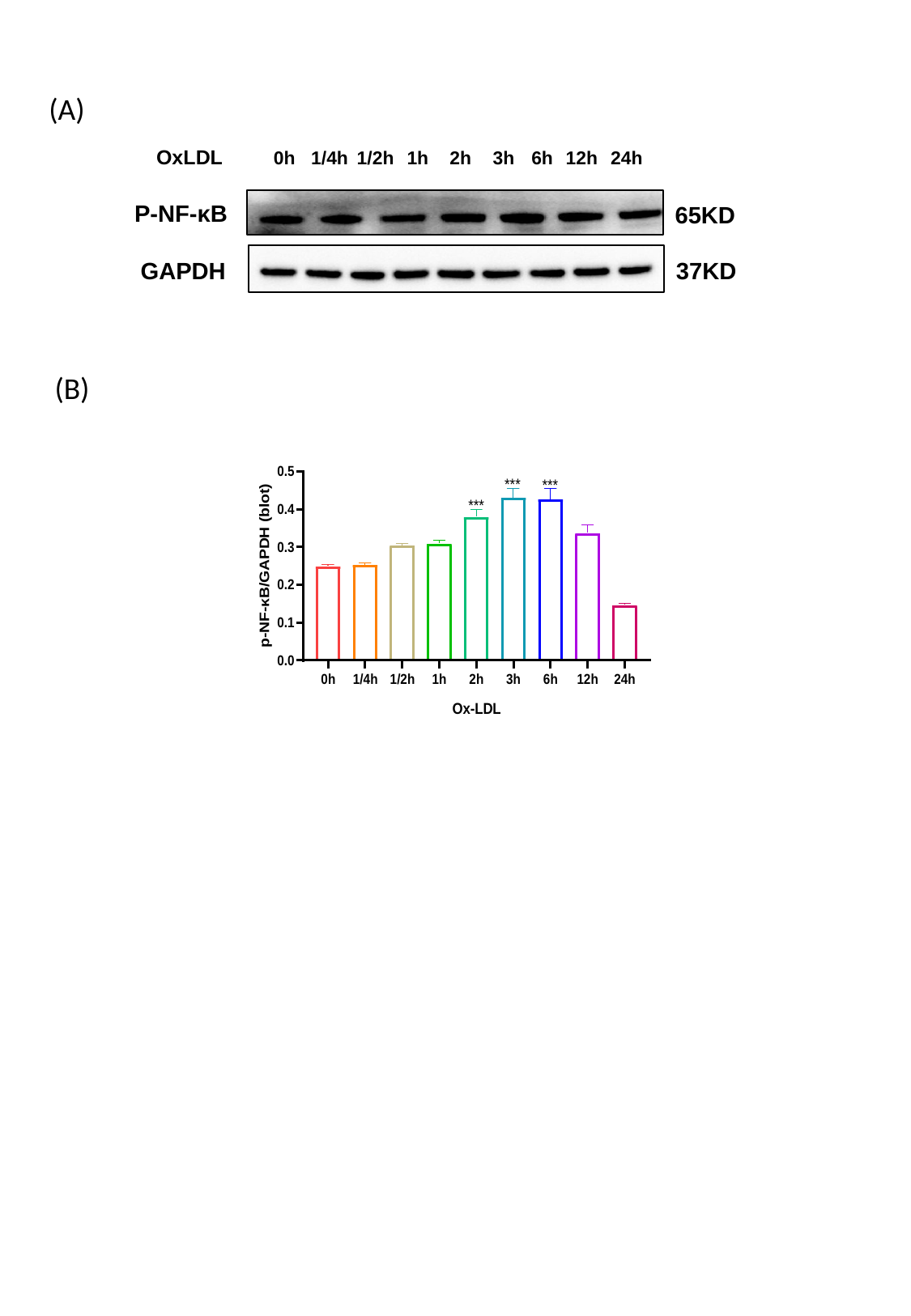

(A)
 OxLDL 0h 1/4h 1/2h 1h 2h 3h 6h 12h 24h
P-NF-κB
65KD
37KD
GAPDH
(B)

## Slide 3
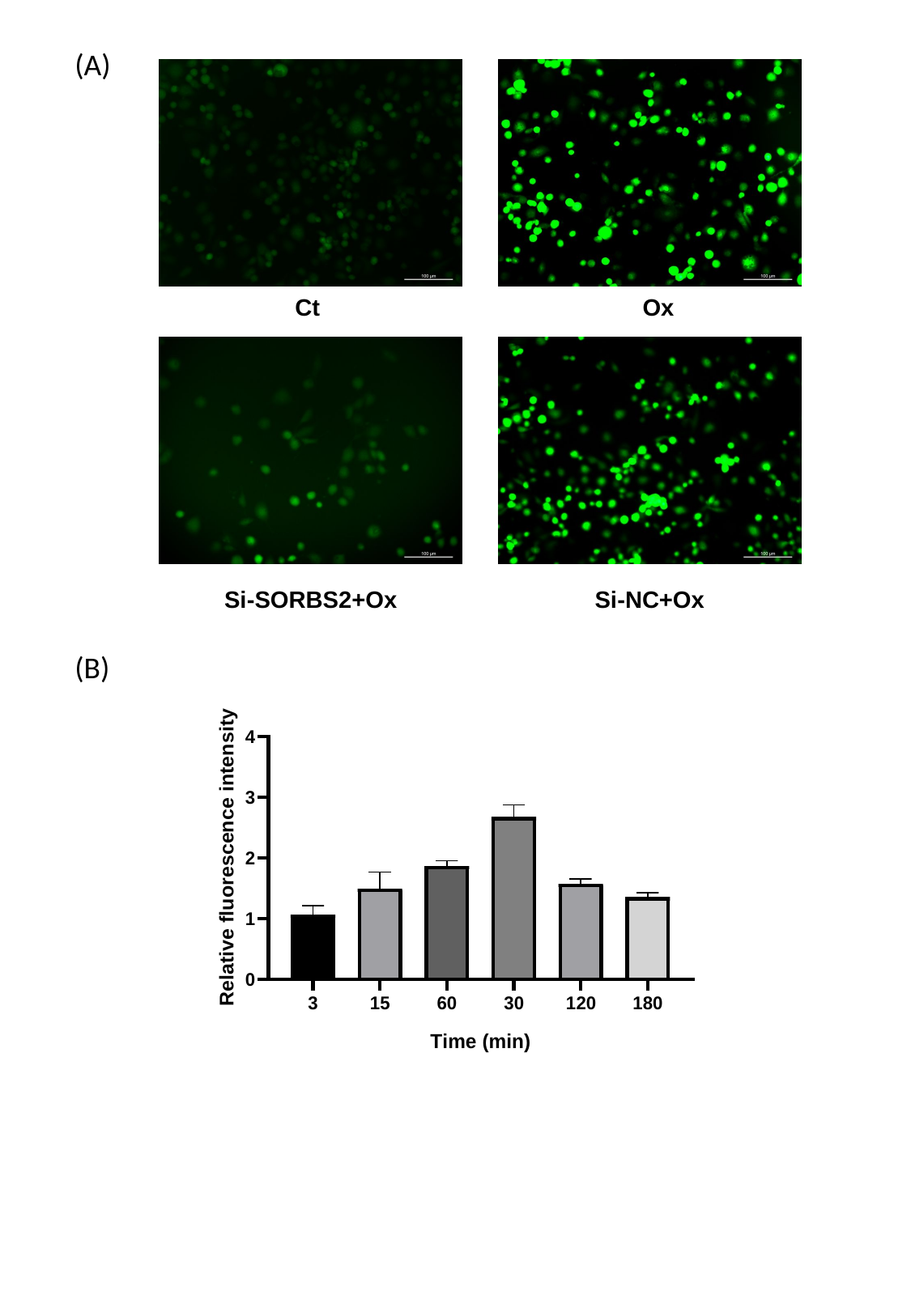

(A)
Ct
Ox
Si-NC+Ox
Si-SORBS2+Ox
(B)

Supplement: Supplementary file 1 — Additional file 1: Figure S1. ELISA results showing concentrations of (A) IL-1β, (B) IL-18, (C) IL-8, and (D) TNF-α in serum from FH patients and normal control participants (n=30). Spearman correlation analyses demonstrated that SORBS2 levels were not significantly associated with (E) HDL-C; and (F) TG. Figure S2. The expression of NF-κB phosphorylation after stimulation upon ox-LDL at different time using western blotting analysis (n=3) (A–B) Macrophages were treated with Ox-LDL (50 μg/mL) for 15min, 30min, 1h, 2h, 3h, 6h, 12h, 24h. Then we collected the proteins at different time points and did western blot analysis of phosphorylated NF-κB. Figure S3. Production of ROS were measured using a Reactive Oxygen Species (ROS) Detection Assay Kit. (A) The cells were observed using fluorescence microscope. (B) Relative fluorescence intensity at different time points were measured using a fluorescence microplate reader (n=3). [file 12967_2022_3381_MOESM1_ESM.pptx]

## Slide 1
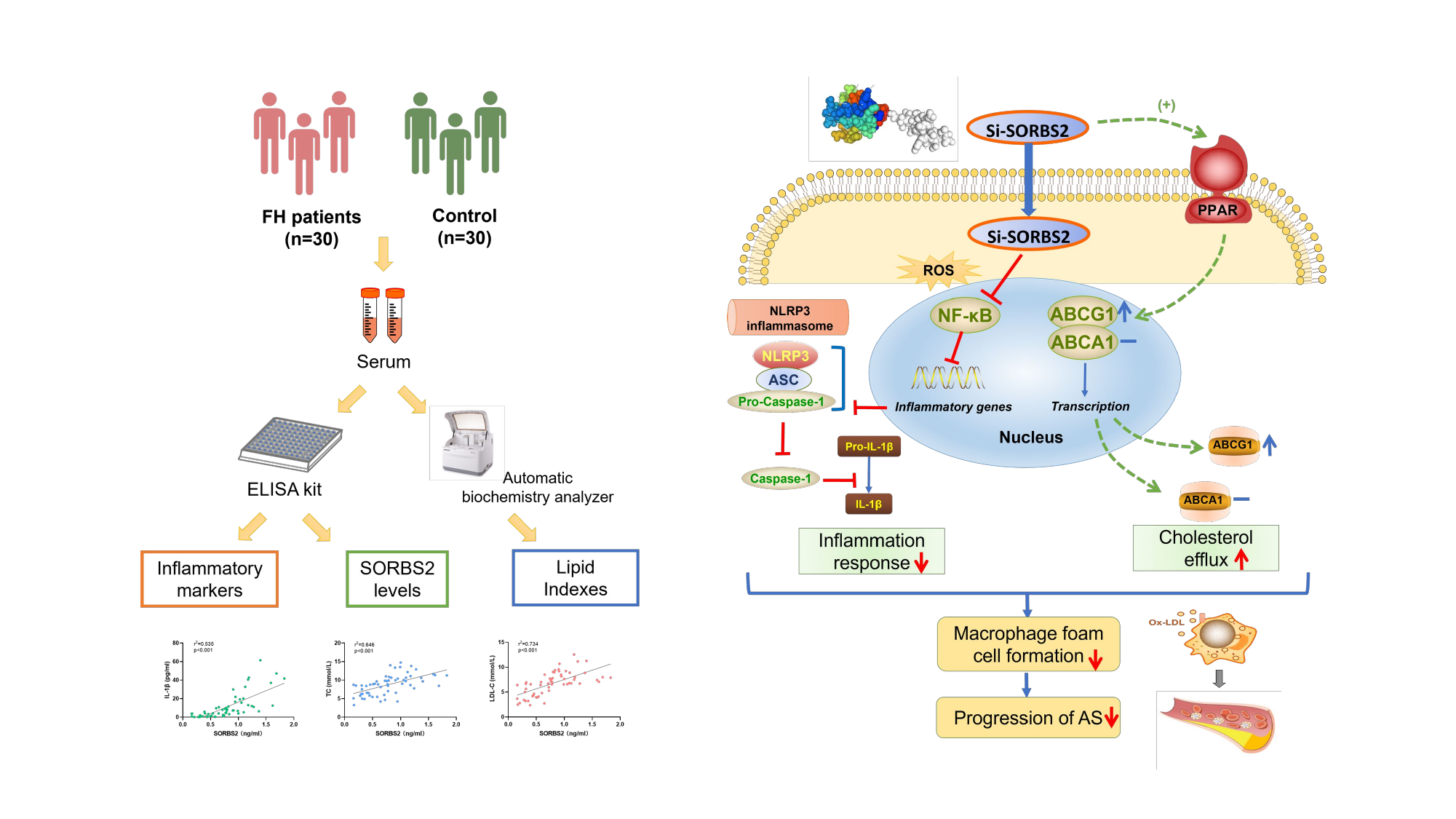

Supplement: Supplementary file 3 — Additional file 3: Central illustration. [file 12967_2022_3381_MOESM3_ESM.pptx]
