# Supplementary material for: SORBS2 as a molecular target for atherosclerosis in patients with familial hypercholesterolemia
Source: J Transl Med. 2022 May 19;20:233. doi: 10.1186/s12967-022-03381-z (PMC9118763; doi:10.1186/s12967-022-03381-z)
Supplement: Supplementary file 2 — Additional file 2: Table S1. Top 20 upregulated overlapping DEGs in GSE6054 and GSE6088. [file 12967_2022_3381_MOESM2_ESM.docx]

**Supplementary Table1.** Top 20 upregulated overlapping DEGs in GSE6054 and GSE6088

| **No.** | **symbol** | **ID** | **adj.P.Val** | **Log2FC** | **Gene.title** |  |  |
| --- | --- | --- | --- | --- | --- | --- | --- |
| 1 | CAPN13 | 229499_at | 0.001 | 1.224782 | calpain 13 | |  |
| 2 | FBXL18 | 220896_at | 0.00316 | 1.109656 | F-box and leucine rich repeat protein 18 | | |
| 3 | PPP1R3A | 206895_at | 0.00386 | 1.721701 | protein phosphatase 1 regulatory subunit 3A | | |
| 4 | GLS2 | 1564706_s_at | 0.00387 | 1.672843 | glutaminase 2 | |  |
| 5 | TRPM3 | 1555252_a_at | 0.00398 | 1.068444 | transient receptor potential cation channel subfamily M member 3 | | |
| 6 | MEOX2 | 206202_at | 0.00399 | 1.983007 | mesenchyme homeobox 2 | | |
| 7 | IGFBP7-AS1 | 1557395_at | 0.00407 | 1.809382 | IGFBP7 antisense RNA 1 | | |
| 8 | SERPINB13 | 211361_s_at | 0.00466 | 2.037063 | serpin family B member 13 | | |
| 9 | HMGA2 | 1568287_at | 0.00483 | 1.956605 | high mobility group AT-hook 2 | | |
| 10 | AGFG2 | 222362_at | 0.00554 | 1.286162 | ArfGAP with FG repeats 2 | | |
| 11 | RUNX1T1 | 242845_at | 0.00557 | 1.179158 | RUNX1 translocation partner 1 | | |
| 12 | SORBS2 | 233720_at | 0.00781 | 1.273496 | sorbin and SH3 domain containing 2 | | |
| 13 | DIAPH3-AS1 | 1555995_a_at | 0.009 | 1.414638 | DIAPH3 antisense RNA 1 | | |
| 14 | SLC5A12 | 214389_at | 0.00935 | 1.636725 | solute carrier family 5 member 12 | | |
| 15 | GULP1 | 215913_s_at | 0.00993 | 1.212085 | GULP, engulfment adaptor PTB domain containing 1 | | |
| 16 | TFAP2B | 215686_x_at | 0.0105 | 1.212736 | transcription factor AP-2 beta | | |
| 17 | ETV3 | 214480_at | 0.011 | 1.02683 | ETS variant 3 | |  |
| 18 | KANK3 | 213715_s_at | 0.0126 | 1.155064 | KN motif and ankyrin repeat domains 3 | | |
| 19 | CELF5 | 232416_at | 0.013 | 1.349483 | CUGBP, Elav-like family member 5 | | |
| 20 | UTS2 | 220784_s_at | 0.0137 | 2.184473 | Urotensin2 | | |

**Supplementary Table2.** Top 20 downregulated overlapping DEGs in GSE6054 and GSE6088

| **No.** | **symbol** | **ID** | **adj.P.Val** | **logFC** | **Gene.title** |
| --- | --- | --- | --- | --- | --- |
| 1 | FAM184B | 233823_at | 0.000329 | -1.71162 | family with sequence similarity 184 member B |
| 2 | CASP2 | 208050_s_at | 0.00108 | -1.05407 | caspase 2 |
| 3 | HELZ2 | 232517_s_at | 0.00132 | -1.47298 | helicase with zinc finger 2 |
| 3 | ANLN | 1552619_a_at | 0.00183 | -1.78683 | anillin actin binding protein |
| 5 | EPHA2 | 203499_at | 0.00254 | -1.65919 | EPH receptor A2 |
| 6 | PVR | 239918_at | 0.00261 | -1.50775 | poliovirus receptor |
| 7 | FGFR2 | 208228_s_at | 0.00272 | -1.22676 | fibroblast growth factor receptor 2 |
| 8 | METTL6 | 233581_at | 0.00313 | -1.66375 | methyltransferase like 6 |
| 9 | FAM107A | 207547_s_at | 0.00411 | -1.22725 | family with sequence similarity 107 member A |
| 10 | RBMS3 | 235570_at | 0.00448 | -1.51394 | RNA binding motif single stranded interacting protein 3 |
| 11 | ZNF311 | 236551_at | 0.00497 | -1.32739 | zinc finger protein 311 |
| 12 | CLEC7A | 1555213_a_at | 0.00517 | -1.09093 | C-type lectin domain family 7 member A |
| 13 | ANKRD18A | 244297_at | 0.0055 | -1.5907 | ankyrin repeat domain 18A |
| 14 | GRIK2 | 1555375_at | 0.00587 | -1.67669 | glutamate ionotropic receptor kainate type subunit 2 |
| 15 | SRC | 1565080_at | 0.00784 | -1.42973 | SRC proto-oncogene, non-receptor tyrosine kinase |
| 16 | LRRN4 | 1553169_at | 0.00816 | -1.47029 | leucine rich repeat neuronal 4 |
| 17 | FCAR | 211307_s_at | 0.00825 | -1.25302 | Fc fragment of IgA receptor |
| 18 | PGM5P2 | 1560431_at | 0.00846 | -1.36967 | phosphoglucomutase 5 pseudogene 2 |
| 19 | MYO10 | 236718_at | 0.00945 | -1.20045 | myosin X |
| 20 | ARHGEF28 | 232994_s_at | 0.0118 | -1.39852 | Rho guanine nucleotide exchange factor 28 |
